# Non-clinical studies in the process of new drug development - Part II: Good laboratory practice, metabolism, pharmacokinetics, safety and dose translation to clinical studies

**DOI:** 10.1590/1414-431X20165646

**Published:** 2016-12-12

**Authors:** E.L. Andrade, A.F. Bento, J. Cavalli, S.K. Oliveira, R.C. Schwanke, J.M. Siqueira, C.S. Freitas, R. Marcon, J.B. Calixto

**Affiliations:** Centro de Inovação e Ensaios Pré-clínicos, Florianópolis, SC, Brasil

**Keywords:** Non-clinical studies, GLP studies, Safety, Pharmacokinetics, Toxicology

## Abstract

The process of drug development involves non-clinical and clinical studies. Non-clinical studies are conducted using different protocols including animal studies, which mostly follow the Good Laboratory Practice (GLP) regulations. During the early pre-clinical development process, also known as Go/No-Go decision, a drug candidate needs to pass through several steps, such as determination of drug availability (studies on pharmacokinetics), absorption, distribution, metabolism and elimination (ADME) and preliminary studies that aim to investigate the candidate safety including genotoxicity, mutagenicity, safety pharmacology and general toxicology. These preliminary studies generally do not need to comply with GLP regulations. These studies aim at investigating the drug safety to obtain the first information about its tolerability in different systems that are relevant for further decisions. There are, however, other studies that should be performed according to GLP standards and are mandatory for the safe exposure to humans, such as repeated dose toxicity, genotoxicity and safety pharmacology. These studies must be conducted before the Investigational New Drug (IND) application. The package of non-clinical studies should cover all information needed for the safe transposition of drugs from animals to humans, generally based on the non-observed adverse effect level (NOAEL) obtained from general toxicity studies. After IND approval, other GLP experiments for the evaluation of chronic toxicity, reproductive and developmental toxicity, carcinogenicity and genotoxicity, are carried out during the clinical phase of development. However, the necessity of performing such studies depends on the new drug clinical application purpose.

## Introduction to Good Laboratory Practice: history and needs for implementation

The formal concept of “Good Laboratory Practice” (GLP) was launched in the USA, during the 1970s, thanks to constant discussions about the robustness of the non-clinical safety data submitted to the FDA for New Drug Applications (NDA). At that time, inspections performed in the laboratories, revealed:

• Inadequate planning and flaws in studies execution;

• Insufficient documentation of methods, results and even fraudulent data (for example, the replacement of dead animals during a study by other animals not properly treated by the test article), which were not documented;

• Use of hematological data from other studies as a control group;

• Exclusion of data concerning macroscopic observations (necropsy);

• Raw data changes in order to "adjust the results" for the final report;

These observations and deficiencies became public and, with the political effect of these claims, the FDA published the first proposals for regulation in 1976 and the final rules in 1979 (1). The GLP principles were the basis for ensuring that the reports submitted to the FDA fully and reliably reflected the experimental work conducted. For the registration of pesticides, the US Environmental Protection Agency (EPA) found similar problems with the quality of the studies. Thus, the EPA published its own regulation draft in 1979 and 1980, and later, in 1983, the final rules were published in two separate parts – insecticides, fungicides and rodenticides (2) and control of toxic substances (3), reflecting their different legal bases.

In Europe, the OECD (Organization for Economic Co-operation and Development) established a group of experts to formulate the first GLP principles. This was an attempt to:

• Avoid non-tariff barriers to the marketing of chemicals;

• Promote mutual acceptance (among member countries) of non-clinical safety data;

• Eliminate the unnecessary duplication of experiments.

The initial proposals were subsequently adopted by the OECD Council in 1981, through the "Decision related to mutual acceptance of data in the assessment of chemicals". The Data Mutual Acceptance (DMA), recommended by the OECD, states that "the data generated in the testing of chemicals in an OECD member country, performed in accordance with the guidelines and GLP principles, should be accepted in other OECD member countries to perform the evaluation and other uses related to the protection of man and the environment". In the following years, several workshops were held in order to improve the content and interpretation of the proposed principles. The result of these meetings was the publication of a consensus or guideline (to support the experimental development). After 15 successful years, the GLP principles published by the OECD were reviewed by groups of experts and adopted by the OECD Council in 1997 (4). Internationally, adherence to the GLP principles is a prerequisite for the mutual acceptance of data generated in a study. Several OECD member countries have incorporated the GLP principles in their legislation.

To facilitate mutual validation, in 1997 the OECD Council adopted the "Adherence of non-member countries to the Council Acts related to the mutual acceptance of data in the assessment of chemicals", in which non-member countries have the possibility of voluntarily adhering to the established standards and, following satisfactory implementation, are allowed to be a part of the OECD program. This required the establishment of national control procedures, and according to which, the authorities of each country should exchange information on the compliance of inspected test facilities and also provide input on the procedures for compliance control. In countries with no officially recognized authorities, individual studies performed by the pharmaceutical industry, in which non-clinical safety data are already developed under GLP standards, can be monitored by foreign GLP inspectors (5).

### Good Laboratory Practices in Brazil

In Brazil, the requirement for GLP began when the Brazilian Institute of Environment and Renewable Natural Resources (IBAMA), by Decree No. 139 of December 21, 1994, established that studies that aimed to assess the potential environmental hazard of pesticides and other chemicals, based on toxicological, ecotoxicological and physical-chemical studies, for the registration and marketing of these products in the country, should be performed by laboratories accredited by the National Institute of Metrology, Quality and Technology (INMETRO), in accordance with the GLP principles (6). As a result, in 1995, the document "Principle of Good Laboratory Practice", with reference to the document published by the OECD called Series on Principles of GLP and compliance monitoring, was published by INMETRO. In 1996, the Decree No. 139/94 was replaced by IBAMA Ordinance Decree No. 84/96. The main idea, however, that laboratories performing the tests for pesticide registration purposes should be accredited by INMETRO, was maintained (7,8).

In 1997, INMETRO and IBAMA established together the criteria for "accreditation", which was replaced in 2010 by the term adopted until today: "recognition of compliance with the Principles of GLP", which is a confirmation by the Accreditation General Coordination (AGC) of a test facility adherence to the Principles of GLP and its inclusion in the Brazilian Program of Conformity with GLP. Thus, in 1998, INMETRO and IBAMA started to create suitable conditions for Brazil’s adhesion to the OECD recommendations, allowing results generated in Brazil to be validated by OECD member countries. In 2009, by Decree No. 220, INMETRO appointed AGC as the Brazilian Compliance Monitoring Authority for the Principles of GLP. In 2011, through AGC, Brazil obtained full adherence to the OECD acts on mutual acceptance of GLP data for the evaluation of chemicals, pesticides, their components and related products. Brazil's full adherence, in a straight vision, means that the results generated in Brazilian recognized laboratories could be automatically accepted by other member countries. This was an important step for the inclusion of Brazil in the worldwide pharmaceutical scenario. For medicines regulation, GLP preclinical studies are the initial part of a long and complex multistep process, without which the release of chemicals for human use should definitely not be allowed.

In other words, Brazilian Test Facilities, recognized by the AGC, started to have tests performed with substances accepted by OECD member and non-member countries with full adherence to Mutual Acceptance of Data (MAD). Other substances, such as pharmaceuticals, cosmetics, food and food additives, veterinary products, sanitizers, genetically modified organisms, among others, tested in AGC recognized test facilities in compliance with the GLP principles, are still not covered by the MAD system. Therefore, other countries have no obligation to accept the tests performed in Brazil with these substances, even if recognized by AGC (6).

### Concepts in Good Laboratory Practice

The concept given the term GLP can be considered an example of concise and precise definition, which not only defines GLP as a quality program, but differentiates it from other systems. GLP restricts actions to organizational processes, where all steps related to non-regulated clinical trials should be developed, from conception and design to the final stages (preparation of the final report and archiving). Thus, GLP is defined as "a quality program related to organizational processes and conditions where non-clinical health and environmental safety are planned, performed, monitored, recorded, reported and archived."

GLP is based on three main figures:

• Test Facility Management (TFM): the person(s) with the authority and formal responsibility for the organization and good functioning of the operational unit in accordance with the principles of GLP. They are responsible, among other functions, for the approval of all operational procedures and the appointment of other figures in the GLP. The mention of management as the first pillar of the principles of GLP is not a coincidence. It is known that the quality of the program will only be successful if it is an internal conviction of the TFM. It is not sufficient to make declarations of conformity that exalt the virtues of quality if incorrect or non-validated information is transmitted to employees, in regard to compliance with and adherence to GLP principles. This includes, for example, omitting when procedures are not accomplished, reducing investments required for compliance with the principles, and not attending the quality unit requirements when the requested changes require greater investment, among others. The collaborators can conclude that only the appearance is important, rather than genuine compliance, which could compromise the entire system (for detailed TFM responsibilities, see section II of the NIT DICLA 035 (9)).

• Quality Assurance Unit (QAU): the QAU is an internal system designed to ensure that the GLP principles are met and that the studies conducted in the installation test are in accordance with these principles. For this purpose, it is a prerequisite that the QAU has independence. Any activity delegated to the QAU cannot compromise its operation and no member of the QAU can be involved in the experimental development, unless they are monitoring functions. It is also essential that the person responsible for the QAU has direct access to different levels of management, in particular the TFM. It is the obligation of the person responsible for the QAU to highlight any deviation or non-compliance with the principles of GLP in any part of the test facility or in any procedure, so that corrective actions can be established (for detailed information on the QAU functions, verify section II of the NIT DICLA 035 (9).

• Study Director (SD): the SD is the only point of control of a study and the only one who supports the study, since they are responsible for the study from the beginning to the end. Thus, the SD ensures that scientific, regulatory and administrative aspects of the study are completely controlled. The SD is usually the researcher responsible for the preparation and approval of the study plan, as well as the data collection and/or its supervision, analysis, reporting and conclusions of the study. The SD has the formal assignment of acting in accordance with the GLP principles, and must prioritize the scientific standard of the studies related to the quality/efficacy of the experimental design, evaluation and significance of the generated data (for more details on the DE responsibilities, see the section II of the NIT DICLA 035) (9).

Although not a requirement, the basic concepts of the GLP principles encourage the appropriate application of science, since the need to prepare a study plan with detailed arguments about the reasons for performing the study, as well as producing the proposal, can certainly lead to a more rational execution of the study. An example to be mentioned among the complex relationships between the GLP and science may be the determination of glucose levels in biological samples. There are complex and highly accurate methods and also simple methods that can only identify the presence or absence of glucose in a biological sample. Any method can be applied in accordance with the principles of GLP (if performed according to the standard operating procedure (SOP) and by allowing its reproduction). However, it is clear that according to the accuracy level required by the scientific proposal of the study, it is the regulatory agency’s role to reject any study which has methodologies that are unable to produce results with the required precision, reliability and reproducibility.

Thus, the GLP requirements are primarily implemented to ensure the quality and integrity of the data and are not directly related to scientific aspects, but to the application of its principles; however, the scientific aspects should be taken into consideration (10).

The regulatory authority can evaluate the data from a study in two ways: i) repeating all experiments, or ii) rebuilding, step-by-step, all activities and circumstances that led to the outcome of the study. Although the first method is the most reliable, it is impractical due to the high cost; in addition, this often violates ethical principles, since it involves repeating previous studies and submitting more animals to toxicity studies. In turn, the second method, despite not providing direct confirmation of the results, implies trust in the data generated, simply because the planning and performance of the experiment, as well as the recording and reporting of data, can be traced and evaluated (if the work performed in the test facility can be considered reliable) (10).

Primarily, the GLP has been developed to combat fraud in the generation and reporting of safety data. However, the goal of the GLP is much broader, as it is not only a control mechanism allowing regulatory agencies to judge the integrity of a study. The principles of the GLP are designed as a tool that also allows improvements in the study and data quality, by applying strict requirements regarding documentation, providing the ability to rebuild any activity, and making the way back to its inception (10).

The GLP requirements are related to several issues and are aimed at different organizational levels of a test facility. Many of these requirements can be considered "common sense" and should be followed when working under the principles of GLP. Broadly, the requirements can be summarized in three points that are the central ideas in GLP:

• Reproducibility: in general, this is the possibility of a third party rebuilding the full course of a safety study, even a long time after its completion, and even in the absence of those who were actively involved in the conduct of their study. This reconstruction capacity is the guarantee that the regulatory agency will need to prove that there were no major faults in the conduct of the study; for example, that all animals received the correct dose of the test article during the entire duration of the study, the correct samples were collected and analyzed, and the compilation of results faithfully reflects the data collected. This provides assurance that the experiments were conducted as described in the report submitted to the regulatory agency.

• Responsibility: this is closely related to the first term. The documentation required to conduct a study, according to the principles of GLP, will report who did what and who can be held responsible for likely errors. On the other hand, if there are any questionable cases, it is also possible to charge the correct person, if they are still employed in the test installation.

• Awareness: the principles of GLP raise awareness of broad tasks, such as administrative work, which is aimed at the quality and transparency of studies conducted in the test facility. The SDs perform the studies under their control in an orderly manner, whilst also raising awareness of achieving small routine tasks, which in theory can be considered dangerous by not requiring "double attention"; if they are not archived, they may culminate in a failure to observe significant effects.

All of these points require general principles to be followed; e.g., that not only are records generated for each activity, event and/or condition, but that they are also kept in an orderly manner to allow the full recovery of the information, whenever necessary.

## Study of distribution, metabolism and pharmacokinetics (DMPK) of new substances

The main characteristics that determine the success of a drug candidate are directly related to its kinetic properties. In this context, through non-clinical studies that are planned and properly executed, it is possible to characterize the pharmacokinetic profile of a substance aiming to establish an adequate dosage that enables patient adherence to treatment and the correct interpretation of results obtained from efficacy and safety studies. Mostly, the toxic effects of substances leading to the discontinuation of their development are associated with prolonged systemic drug exposure, the formation of toxic/reactive metabolites and/or possible drug-drug interactions (11). A number of drugs such as troglitazone, trovafloxacin and bromfenac (oral formulation) were withdrawn from the market due to the formation of reactive hepatotoxic metabolites (12) and other drugs that are still on the market such as acetaminophen and amiodarone, etc., can cause hepatotoxicity when used at high doses (12).

Considering the idea that the pharmacokinetic properties of a substance are determinant to its success in clinical studies, the pharmaceutical industry has introduced DMPK (Drug Metabolism and Pharmacokinetic) studies in the early phases of new drug development. A recent study performed by four important pharmaceutical companies (AstraZeneca, UK; Eli Lilly and Company, USA; GlaxoSmithKline, UK; and Pfizer, USA) showed that, with the introduction of DMPK studies during the lead identification phase, the pharmacokinetics represent only 5% of the reasons for failure in clinical development (13). From the financial point of view, the discontinuity of a project during the final phases generates losses of around 90% of the total investment (14). Therefore, the DMPK studies carried out during the non-clinical phases are extremely important, since they reduce the time and costs expended with the development of new drugs. In addition, the DMPK performed during the early phases of drug development provide important information about the structure of a molecule and possible modifications that can be made in order to optimize its DMPK properties.

Finally, data about the pharmacokinetic properties of a new substance together with preliminary studies about its safety and efficacy are critical to take the decision to continue or not (go/no-go decision) the development process of a new drug (15).

## Factors that determine the pharmacokinetic profile of substances

### Physical-chemical characteristics and physiological properties

Several physical-chemical properties such as lipophilicity, rate of dissolution, solubility, pKa and molecular weight, can directly interfere with the absorption, distribution, metabolism and elimination of a substance (16,17). The lipophilicity and rate of dissolution are expressed as LogP (partition coefficient of non-ionic substance between the hydrophilic and lipophilic phase in water/octanol system) and LogD (partition coefficient of ionized substances, normally weak acids and bases) (17). Substances with LogP>5 are considered highly lipophilic and, although presenting high membrane permeability, they also show low solubility which hampers their absorption (16).

The solubility of a substance in different physiological conditions, as well as the lipophilic characteristic, also interferes with its absorption. One of the factors that directly influences the solubility of a substance is its pKa (the negative base-10 logarithm of the acid dissociation constant (Ka) of a solution). It defines the concentration at which neutral and ionized species of a molecule are equally distributed in a specific pH (17). The knowledge related to the solubility and permeability of a substance is a very important aspect since it allows the prediction of bioavailability after oral administration and its classification according to the Biopharmaceutical Classification System (BCS) (for details about BCS, see 18,19).

The absorption rate of a substance after oral administration depends mainly on its capacity to cross the intestinal epithelium. Different *in vitro* methods are used to determine the intestinal permeability of a substance, such as: i) human colon adenocarcinoma cells (Caco-2); ii) Madin-Darby canine kidney (MDCK) cells, and iii) the parallel artificial membrane permeability assay (PAMPA). However, Caco-2 cells are considered the gold-standard assay for permeability evaluation of substances that were developed for the oral route of administration due to its spontaneous differentiation process, which leads to the formation of an enterocyte monolayer with preserved morphological and functional characteristics (15,17 [Bibr B18] [Bibr B19]). Besides the physical-chemical properties, physiological factors associated with the binding affinity to plasma proteins and the metabolic stability of a substance are also important to determine its DMPK properties (11,20).

The binding of a substance to plasma proteins, mainly to albumin and acid α1-glycoprotein, occurs quickly and reversibly until it establishes the kinetics equilibrium between the bound and unbound form. Only the unbound form is able to cross the capillary and reach the target organ. The concomitant administration of more than one substance could interfere with the binding affinity, producing rather an exacerbated pharmacological effect or not producing the desired therapeutic effect. The binding evaluation of a substance to plasma proteins can be performed using *in vitro* experiments by means of ultrafiltration techniques and equilibrium dialysis (11).

The metabolization process of a substance acts as a body’s defense system, leading to the modification of foreign substances (xenobiotics) by chemical processes to promote their elimination from the organism (20). The biotransformation studies allow evaluation of the metabolic stability level of a substance and the prediction of possible metabolites’ formation, which are more active than the parent substance (prodrug), or even toxic metabolites; it also allows evaluation of whether the parent substance, which is often metabolically unstable, can reach the therapeutic concentration that is necessary to produce the pharmacological effect (15,20).

The main organ in the body responsible for metabolization and detoxification of substances is the liver. It can also occur in the lung, kidneys, gut and blood plasma. Drug-metabolizing enzymes of the system cytochrome P450 (CYP450), mainly CYP1A2, CYP2C9, CYP2C19, CYP2D6 and CYP3A4 are responsible for phase I reactions (oxidation, reduction, hydrolysis, dealkylation, and deamination), which promote the conversion of a lipophilic compound to more hydrosoluble metabolites, which are easily eliminated from the human and/or animal body (15,20). CYP3A4 is the most abundant cytochrome P450 isoform in human liver and has a broad substrate specificity. CYP3A4 is involved in the metabolism of almost 50% of all drugs available on the market (20). To determine the metabolic stability of a substance, both *in vitro* and *in vivo* tests should be performed. The cellular systems most often used for *in vitro* metabolic stability studies are: i) liver microsomes; ii) S9 fraction, and iii) culture of human hepatocytes for the determination of hepatic clearance (15,20,21).

The identification of the main enzymes responsible for the metabolization of a substance *in vitro* generates detailed information about its metabolization process. It allows adequate guidance for the clinical study related to: i) substance interaction; ii) dose selection (to those patients with kidney or liver damage), and iii) toxic effect prediction (22–24). In parallel, the assay performed in microsomes that uses liver samples from humans and from other species should be conducted to evaluate possible inter-species differences. Such results will indicate which species is more appropriate to execute the toxicity studies, as well as to evaluate the possible involvement of the CYP3A4 and CYP2D6 enzymes in humans (15,20,21).

Several medicines were withdrawn from the market due to their interaction with other substances (mainly related to the CYPs) for example: Seldane^®^ (Terfenadine, Aventis Pharmaceuticals, USA), Posicor^®^ (Mibefradil, Roche, Switzerland), Propulsid^®^ (Cisapride, Janssen-Ortho, Canada), Lotronex^®^ (Alosetron, Prometheus Laboratories Inc., USA), Baycol^®^ (Cerivastatin, Bayer A.G., Germany) and Serzone^®^ (Nefazodone, Bristol-Myers Squibb, USA) (25). There are several examples of drugs that induce CYPs or, in other words, increase the metabolization process, for example, barbiturates, rifampicin, omeprazole and alcohol. On the other hand, drugs such as quinidine, ketoconazole and sulphaphenazole inhibit the CYPs reducing the metabolization process (21–23). Besides the enzymes cited above, phase II enzymes (transferases), such as sulfotransferase, glucuronyl transferase and glutathione-S-transferase, enhance xenobiotic elimination based on the conjugation reactions. Other enzymes are also involved in the chemical process of biotransformation, such as alcohol dehydrogenase, aldehyde dehydrogenase, and NADPH-quinone oxidoreductase (22,23). Thus, the identification of enzymes responsible for the process of drug metabolization is recommended by regulatory agencies during the discovery and development phases to evaluate possible drug-drug interactions (15,22).

### Determination of pharmacokinetic properties *in vivo*

The *in vivo* pharmacokinetics assays allow the quantitative evaluation of the time course of absorption, distribution, metabolism and elimination (ADME) of a new substance; this information is very useful for predicting the desirable dosage and the appropriate posology protocol to be used (15).

Initially, during the non-clinical phase, performing exploratory pharmacokinetic studies is suggested. These studies aim to support the pharmacology assays, the interpretation of toxicology and efficacy studies, and dosage selection and the compound/drug formulation optimization. During this phase, a reduced number of animals are used and blood samples are collected until 6 h after the administration of substances. A limited volume can be collected in accordance with the species and animal weight. For more details about the recommended blood volume to be collected, please see “Guidelines for Survival Bleeding of Mice and Rats” developed by the National Institutes of Health (26). It is recommended to follow these limits since excess blood withdrawal can interfere with the pharmacokinetics profile of the substance.

From initial pharmacokinetic (PK) screening, it is possible to select substances that show adequate PK properties and, after this, the selected substances are submitted to complete screening. The screening models currently used are snapshot PK, rapid PK and full PK. However, the strategy choice depends on many factors, including materials and tools available, researchers’ knowledge and definitions of the PK parameters to be analyzed (27).

During the PK profile analysis of a substance, the following parameters should be determined: i) area under the curve (AUC); ii) maximum drug concentration in plasma; iii) time to reach the maximum concentration; iv) half-life; v) distribution volume; vi) clearance; and vii) bioavailability.

## Desirable DMPK properties of a candidate substance intended for oral route administration

A substance intended to be used *via* the oral route should present some critical properties in DMPK studies: i) water solubility; ii) high permeability and low efflux in Caco-2 cells; iii) sufficient bioavailability to reach the desirable plasma and organ to produce the pharmacological effect; iv) adequate half-life time to the intended posology scheme in human; v) linear PK; vi) elimination that is not dependent on a single route or on a single metabolization enzyme, without forming active or reactive metabolites in large amounts and without interacting with metabolization enzymes in relevant concentrations; vii) acceptable safety margin (therapeutic index, preferably higher than 10 times); and viii) established PK-PD (pharmacodynamics) relation (15).

## Toxicokinetic assay

The toxicokinetic assays are usually performed during toxicology studies and should be conducted in accordance with the GLP rules. The toxicokinetic assays measure the systemic exposure of a substance in animals and establish the relationship between the dose administered and the time course of the substance in the toxicity studies. Indeed, the PK profile determination of a substance following administration of multiple doses enables the best interpretation of the toxicological findings. The toxicokinetic study also evaluates the potential of a substance to accumulate in a specific organ and/or tissue. Thus, the data generated with these studies should contribute with the data obtained with the toxicology studies in terms of interpretation of the toxicity tests and in comparison with the clinical data as part of the risk and safety evaluation in humans. The toxicokinetic studies are part of the non-clinical test battery recommended by the regulatory agencies (11,28).

The main questions that should be answered to facilitate the comprehension between the systemic exposure of a test article and the quantification of the absorbed fraction in the tissues are: i) is the substance absorbed?; ii) what is the absorption rate?; iii) how is the substance (parent/metabolite) distributed within the body?; iv) is the substance metabolized?; v) if yes, in which organ/tissue, what is the rate of metabolization, and which metabolites are formed?; vi) what is the route and rate of elimination?; and vii) what is the effect of the dose on absorption, distribution, metabolism, and elimination? (29).

Two protocols can be applied for toxicokinetic studies: a full protocol that aims to answer all aforementioned questions or a reduced protocol, in which only the main questions are answered to corroborate the interpretation of the toxicology findings. In the full toxicokinetic protocol other biological matrices should be collected besides blood, such as excrements (urine and faeces), fat, muscles, liver, kidneys, possible target-organs and skin (when the substance test is administrated by the dermal route). Moreover, if the substance in its parent form and/or its metabolites are volatile, additional animal groups should be included for collecting excrements, carcasses and expired air in order to determine the extension of absorption and the route(s) of elimination of the substance. The design of the study and the selection of the experimental protocol should be defined on a case-by-case basis; overall, they should be able to provide enough information to evaluate the risks and safety of the candidate substance (28,29).

Considering the importance of evaluating PK properties during the development of new drugs, the assays described in Table 1 are highly recommended.

**Table:**
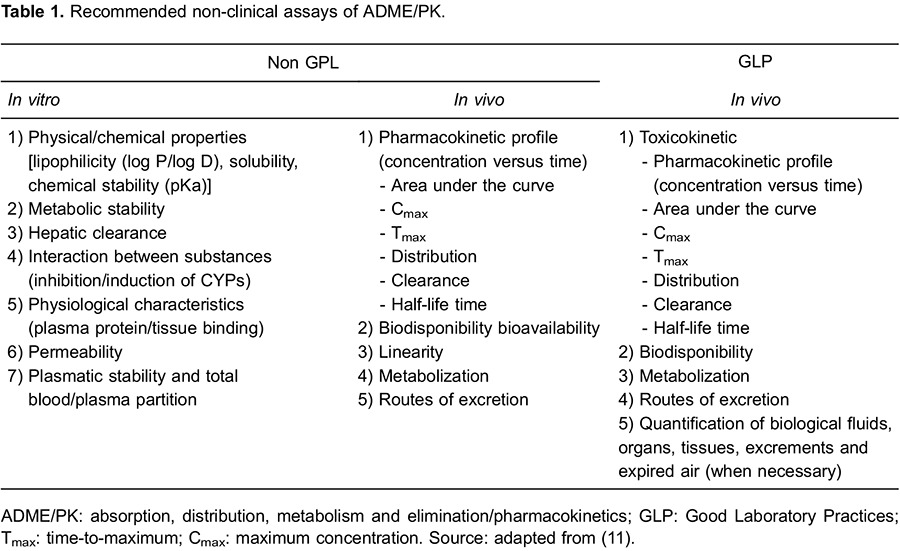

## Chemical characterization, manufacturing and manufacturing control

The chemistry, manufacturing and controls (CMC) of an active/final product are important for the adequate execution of the non-clinical and clinical studies of a candidate as well as for the correct interpretation and correlation between the results obtained in each phase of the discovery and development process. It is recommended that the manufacturing process follows the Good Manufacturing Practices (GMP) in order to guarantee the quality, safety and efficacy of the pharmaceutical products and to ensure the manufacturing consistency and batch-to-batch reproducibility (30).

Since CMC of an active substance/final product provides important information that guarantees its identity as well as its quality during the manufacturing process, the CMC submission to the application for product registration represents one of the requirements of regulatory agencies. More details on the CMC of an active substance/final product requested by regulatory agencies can be found in the guideline M4Q (R1) (31).

## Safety studies

### Safety studies to evaluate toxicity

The recent advance in the development of new drugs has become a challenge for science, as the offer of new therapeutic approaches has required techniques that guarantee its safety in humans. Non-clinical safety studies have been performed based on the experience and employment history in a specific animal species before safety tests have been performed in humans. Besides animal studies, several *in vitro* tests have been developed and validated for safety evaluation to discover the toxicological potential of substances. However, these assays are sometimes complementary to the *in vivo* tests.

The use of animals to evaluate the toxicity of compounds started in 1920, when J.W. Trevan introduced the Lethal Dose 50% (DL50) concept. After this, Food and Drug Administration (FDA) scientists started to develop new methods, such as the ocular and cutaneous irritation in rabbits that were widely accepted and applied all over the world. In addition, the researchers of the National Institute of Cancer in the USA started to develop tests in mouse to predict the cancer-causing potential of new substances. However, after 1960 and due to several births of children with limb deficiency caused by Thalidomide use during pregnancy, safety studies performed initially in animals were required. After those facts, the FDA required an Investigational New Drug (IND) application for all new substances that progress to clinical tests. The IND application must contain the safety and efficacy data of the substance before the first human exposure (see more details below) (32).

At the end of 1980, the OECD and the International Conference on Harmonization (ICH) published guidelines for toxicity in non-clinical tests for chemical and pharmaceutical substances, which are still recommended by the majority of the regulatory agencies. Since its publication, new revisions and assays were implemented throughout the years, aimed at the promotion of more predictive and ethical tests that could reduce or even prevent the use of animals.

These guidelines present the basis of how assays should be conducted, suggesting species to be used, duration of the assays, organs to be investigated and the analysis to be conducted, as well as which data should be presented in the final report. Even so, these guidelines are still generating several doubts in the scientific and industrial communities as the assays are not presented in detail.

**Figure 1:**
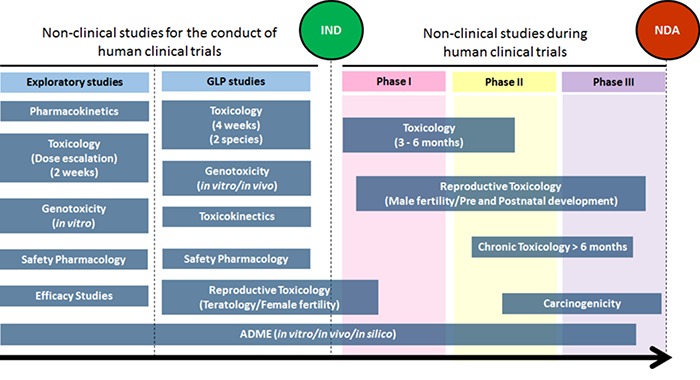
Steps of non-clinical studies in drug development process. GLP: Good laboratory practice; IND: Investigational new drug; NDA: New drug application; ADME: absorption, distribution, metabolism and elimination.

The performance of non-clinical tests in sequence is an important factor in the development of a new medicine. Despite of there is not a standard program to execute the assays, a well-designed planning helps avoiding several errors or unnecessary tests, besides saving time and financial resources. In Figure 1, we suggest the non-clinical studies that should be performed during drug development Thus, in this section, we will discuss the main non-clinical safety tests that are required in the process of drug development, such as mutagenicity tests, acute, sub-chronic and chronic toxicity, developmental and reproductive toxicity, carcinogenicity, local tolerance and safety pharmacology.

#### Preliminary toxicology studies

In the initial phases of development, several promising selected substances follow exploratory safety test screening to assess possible toxic effects. The exploratory tests are normally performed *in vitro*, or with a reduced animal number and do not require conformity with GLP principles. For this reason, these studies present reduced costs in comparison to the GLP studies that are required in subsequent steps in the drug development process. The exploratory assays are essential for the initial decision making regarding the investment on a new substance, since it could provide relevant information, which directly affects the planning of non-clinical assays that will be performed.

One of the initial tests to evaluate the toxicity of a new substance is the preliminary Ames test. This test is performed to evaluate a possible genotoxicity effect and the detection of genetic alterations in organisms exposed to these substances. The different genotoxicity tests detect potential genetic and chromosomal mutations in organisms. The Ames test is an *in vitro* mutation assay in bacteria and has the ability to detect any mutation promoted by the substance, enabling the reversion of the existent bacterial mutation and restoration of the functional bacterial capacity to synthesize an essential amino acid (histidine). This test can either evaluate the mutation capacity of the substance or its metabolites. The execution of this test is mandatory to most of the substances in the drug development process. Despite the test being regulated and required by the authorities, the preliminary assay is fundamental for the early detection of possible genotoxic effects of the test article. Furthermore, the *in vitro* micronucleus assay has emerged as one of the preferred methods for assessing chromosome damage. At this development level, the genotoxicity is restricted to *in vitro* tests; however, in the subsequent steps, other tests of genotoxicity are required, such as the *in vivo* micronucleus test.

The first *in vivo* toxicity study for a new substance is usually a dose range finding (DRF) study in rodents. For both scientific and welfare reasons, it is common practice to explore adverse effects in rodent species prior to non-rodent species. This increases the amount of information available for the design of non-rodent studies; for example, data from the initial rodent study can be used to set the starting dose, or allow specific monitoring of adverse effects in non-rodents. The Maximum Tolerated Dose (MTD) test by dose escalation scheme is a common test used for the dose selection in GLP toxicity studies with a duration of 30 days. The MTD test allows the identification of the dose at which target organ toxicity is likely to be observed, but without further study implications due to the animals’ morbidity and mortality (33).

MTD is defined as the highest dose tolerated in a toxicology study. The methodology is normally determined by parameters such as clinical signals, body weight changes, food consumption, morbidity and mortality. Besides that, several dose selection protocols also recommend the hematological and biochemical analysis execution, as well as the histopathological analysis of target organs to better determine the toxicity between the tested doses.

The acute or repeated-dose toxicity studies can be performed during the preliminary phase of the development process. Although the MTD or dose escalation studies provide important information about drug toxicity, the repeated dose studies have more complete protocols, which consider the histopathology of a set of organs, more complex behavioral and clinical analysis, complete biochemical and hematologic analysis, ophthalmological analysis and groups for the evaluation of the side effects recovery after a treatment period. In the acute toxicity protocol, the effect of a single administration of three different doses is usually evaluated and the animals are observed for 14 days after treatment. The OECD guidelines do not require the acute oral toxicity assay for pharmaceutical products, but some regulatory agencies suggest this assay. Also, according to the M3 guideline (R2) (34), the acute toxicity is only recommended when there are no other studies about toxicity, such as MTD or dose escalation. In this case, the acute toxicity studies can be limited and provide information about the administration routes and the doses to be administered. These data can be collected from non-GLP studies. However, the clinical treatment planning can only be supported by toxicological repeated dose studies performed in accordance to the GLP rules M3(R2) (34).

The short-term repeated-dose toxicity study is another protocol suggested during the exploratory phase. The most indicated test is the repeated dose 28-days oral toxicity study No. 407 (35). Since it evaluates the toxicity level of continuous administration, this test can provide more precise data, although it is more complex in comparison to the acute toxicity and MTD tests. This protocol is normally required for the first exposure of the substance in human (Clinical phase I); however, its execution will depend on the objective of the clinical treatment regime, as its duration in humans is directly related to the non-clinical protocol. It is important to mention that deciding which exploratory or regulated toxicology study will be performed requires deep planning by the development team. Considering that the basis of defining which studies should be performed is related to the intended clinical use of the test article, the interaction between the non-clinical and clinical study teams is required.

#### Regulatory toxicology studies

Regulatory toxicology studies are mandatory in the drug development process and aim to evaluate the toxicity level of a substance using protocols that follow the guidelines recommended to conduct non-clinical studies of pharmaceutical products. In addition, it is important to emphasize that they have to be conducted in compliance with the GLP principles. After the preliminary toxicity studies, the GLP studies should be conducted in two animal species (with the exception of mutagenicity tests). The planning of these studies could be based on the data obtained by the exploratory studies of both efficacy and toxicity. These findings could help to define doses, the duration of study, and any side effects that could require special attention. Some GLP toxicology studies are required before beginning clinical trials, but others could be conducted during different phases of the clinical trials; this will be discussed further in this section.

Although there are no unique and standard plans for drug development, it is recommended to perform genotoxicity studies (*in vitro* and *in vivo*) as well as a study of dose selection and repeated-dose toxicity (28 days), before the first exposure of humans to the substance. Usually, with these studies series, together with pharmacokinetic, efficacy, safety pharmacology and substance chemical characterization studies, it is possible to submit a dossier to the regulatory agencies to request permission to start the tests in humans. It is important to emphasize that for toxicology studies following GLP principles, the test article should be in its final formulation, in other words, in the same formulation that will be used to treat individuals during the clinical studies, together with its complete chemical certificate of analysis. In addition, the route of administration should be, preferably, the same as that intended for human treatment. These requirements are clearly described in the guidelines of non-clinical studies of the main regulatory agencies, such as the FDA, European Medicines Agency (EMA) and Agência Nacional de Vigilância Sanitária (ANVISA, Brazil).

In this step, the genotoxicity tests previously described (*in vitro* Ames No. 471 (4) and *in vitro* micronucleus No. 487 (36)) should be performed in accordance with the GLP requirements, even when it has already undertaken exploratory studies. Thus, the *in vivo* micronucleus test No. 474 (37) is also recommended, as it provides relevant genotoxicity data of a substance involved in active processes, such as metabolization, pharmacokinetics and DNA repair, which are not totally detected by an *in vitro* system. This test evaluates micronucleus formation in erythrocyte samples from the bone marrow or from rodents’ peripheral blood samples, allowing the identification of possible cytogenetic damage, resulting in micronucleus formation and chromosomal alterations. In many cases, the genotoxicity assays, performed according to the GLP principles, are conducted before the repeated dose toxicity tests for decision-making reasons. However, it depends on the strategy programmed for each substance and on the obtained preliminary data. Also, execution of the GLP genotoxicity test concomitant with initial repeated dose toxicity studies is common.

An important decision during the planning of non-clinical studies is the duration of the repeated dose toxicity study that is normally based on the duration, therapeutic indication and planning of the clinical study. Generally, the duration of toxicity studies conducted in two mammalian species (rodent and non-rodent) should be the same or even longer than the studies in humans, but no more than the maximal time recommended by the M3(R2) guideline (34) for each species (see more details in Table 2). This table describes the recommended duration of repeated-dose toxicity studies to support the conduct of clinical trials. The relation between animal and human studies is a very important point in the drug development process, once the conduction and the choice of non-clinical studies should justify the time duration proposed for clinical treatment.

**Table:**
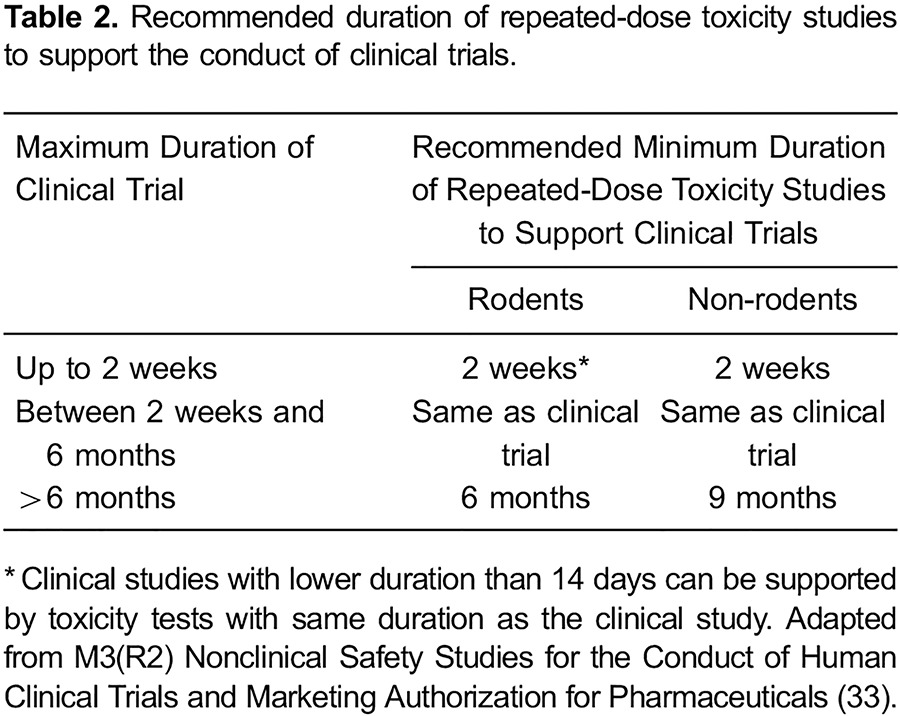

The repeated dose toxicity studies have guidelines with a very well defined duration, such as the guidelines No. 407 (repeated dose 28-days oral toxicity study in rodents), No. 408 (repeated dose 90-days oral toxicity study in rodents), No. 410 (repeated dose 21/28-days dermal toxicity study), No. 452 (chronic studies of toxicity), etc (4,35,36,38–40). One of the most used protocols before the first exposure of substances in humans is the repeated dose for 28 days. As mentioned in the exploratory studies section, this test aims to collect information about possible health risks using the repeated exposure to a substance, including its central effect, and that on the immunological, endocrine and reproductive system.

Although this test is indicated for oral administration, other parenteral administration routes could be used if well justified and if they are similar to the clinical uses. In addition, the toxicity test following repeated doses of the substance could also be applied for 14 days when it is justified by the short time of treatment in the clinical phase. Besides, it is highly recommended to add a recovery group to the study in order to observe possible toxic effects recovery. The repeated dose toxicity study should be performed in accordance with the GLP requirements. The obtained outcomes are fundamental for characterizing the toxicity of the test article and provide a relationship between the dose-response and toxicity data to determine the no observed adverse effect level (NOAEL).

The toxicity data described in the guidelines suggested by the FDA comprise an important basis for the IND application; however, it depends on the intended application of each substance and can vary case-by-case. After the authorization to start clinical studies, other non-clinical toxicity studies should be conducted; for example, sub-chronic and chronic studies. The toxicity evaluation is normally classified in accordance with a chronological scale, such as the acute studies that are performed to verify the substance effect using single or repeated dose administration for 24 h. Indeed, sub-acute studies are those that comprise the toxic effects for 30 days, whereas sub-chronic studies are defined by the toxic effect of a substance between 30 and 90 days. Studies that are superior to 90 days are normally classified as chronic. However, this classification can be specific for some species; for example, chronic studies can be performed for six months in rodents and 9 months in non-rodents (41).

The sub-chronic study (90 days) can be conducted in parallel with phase I clinical studies. This study is very similar to the toxicity study of 28 days, and the guidelines for both require a daily treatment with at least three doses of the substance and the vehicle, together with clinical, biochemical, hematological, anatomical and histological analysis that are detailed in each guideline. Despite standard measurements, some additional analyses could be included for the observation of a particular effect of the substance, mainly when several toxic effects are described. These tests should be conducted in accordance with the GLP principles; together with the clinical data obtained from phase I, these can help to decide whether the study should continue or not to phase II.

The reproductive toxicology test occurs during the clinical studies, along with the teratogenic potential evaluation. The reproductive toxicology test is the most rigorous test applied by the FDA and is a prerequisite for the approval of new substances. In accordance with the guideline S5(R2) (42), the drugs can affect the reproductive activity by:

• Fertility and initial embryo-fetal development (implantation);

• Embryo-fetal development or teratogenicity;

• Pre- and post-birth development, including the maternal function.

The phase I study can often start in voluntary male participants, even without reproduction/development data, as long as the substance has not shown evidence of testicular damage in the studies of repeated doses of 2 to 4 weeks’ duration (43). The requirement of reproductive toxicology studies in the beginning of the clinical phase I may differ in each country; however, it is quite often that these tests require clinical studies involving women of fertile age. The fertility and implantation tests include male (28 days) and female (14 days) treatments with the substance before mating, and are characterized by the semen analysis (counting and viability), number of implanted embryos and survival of the embryos at the sixth day of pregnancy. The embryonic and fetal tests are normally performed in two or three species (rats, mice, rabbits); the substance is administered to females in the initial period of pregnancy (in rats, 6–16 days after mating). In this case, the animals should be euthanized before giving birth, aiming to count the embryo number and observe abnormalities. In the pre- and post-development tests, females are treated during pregnancy and lactation, where the offspring can be observed according to the motor activity after lactation. In these cases, some pups are analyzed according to their abnormalities in different stages of development, even in adulthood, to evaluate their sexual performance and their second offspring (42,43). Despite some *in vitro* assays of reproductive toxicity being routinely performed, they do not provide enough data about teratogenic potential in mammals and are not recognized and required by the authorities (43).

The reproductive test battery is a requirement in the drug development process for almost all regulatory agencies; however, for herbal products, ANVISA suggests that these assays should not be performed. Thus, the following statement should be described in the product instructions: “it should not be used by pregnant women and nursing mothers since there are no studies providing its safety under these conditions” (44).

In addition to the general toxicology and reproductive toxicity studies, the carcinogenicity test is usually required for drugs intended for continuous treatment of 6 months or more. In these cases, carcinogenicity studies should be carried out before the substances go to the market, but never before the beginning of clinical tests. The carcinogenicity assay could be required in case of substances belonging to a known carcinogenic group or when chronic studies of toxicology present consistent evidence of the carcinogenic potential, or even when there is evidence showing that the substance or its metabolites are retained in the organism for a long period (43). Interestingly, in the absence of other data, substances with positive evidence in the genotoxicity tests are considered carcinogenic to humans and are not submitted to long-lasting carcinogenicity tests. However, in case the substance has been used for chronic treatment in humans, chronic tests (for about 1 year) could be necessary to assess possible tumorigenic effects (45).

The carcinogenicity studies are normally carried out during phase II and III of clinical development, using only a rodent species, especially rats. In addition, it is recommended to perform other *in vivo* assays that can provide additional information about the sensitivity of the carcinogenic substance, such as short duration test in transgenic mouse or carcinogenicity test of long duration in other rodent species (mouse). The carcinogenicity study of long duration in rats is usually conducted for at least 2 years of treatment with three or four doses of the test article and the control. Generally, the lowest dose to be tested in these non-clinical studies is the maximal dose recommended in humans, while the highest dose is the MTD obtained in the previous safety studies. To perform the carcinogenicity studies, it is necessary to include 50 to 80 animals per group/gender. This means that the entire study needs around 600 to 800 animals being treated and evaluated for up 2 years. The ICH guidelines (45–48) determine the rules to be followed in these studies, which require the performance of the studies according to the GLP principles, with specific pathogen-free (SPF) animals and the histopathological analysis with more than 50 tissue types being analyzed by a veterinary pathologist with experience in carcinogenesis. The carcinogenicity test is one of the most difficult and expensive studies during the non-clinical developmental process.

Depending on the observed effects in the standard toxicological studies, other tests could be required. For example, if the drug candidate induces alterations in the immunological cells or in the lymphoid system tissues, immunogenicity studies could be necessary. Such studies are performed with substances that act by modulating the immunological system or those causing alterations such as necrosis, apoptosis or interactions with cellular receptors shared by different tissues and non-target immunological cells (46). Some of these evidences could be obtained by hematological, biochemistry and histopathological analysis obtained from previous toxicological studies. In these cases, assays such as the T-cell dependent antibody response (TDAR) test, immunophenotyping, natural killer cellular activity, etc. are recommended (48). Furthermore, for substances previously known as immunogenic, the sensibility test could also be necessary. In addition, for substances that are administered topically, local tolerance tests are required before the beginning of clinical phase I, and could be part of other toxicology studies. This assay aims to evaluate the tolerance level of a substance in different regions of the body with which it could have contact. To perform these tests, the selection of the species depends on each assay type as well as the administration route, the dosage and also the exposure time in accordance with the duration of the study to be conducted in humans. The local tolerance test can include the administration route (dermal, parenteral, ocular, rectal, etc.) and tests of systemic toxicity. The guide CHMP/SWP/2145/2000 Rev. 1 (49) contains the detailed information about each test.

As previously described in this section, the toxicology tests are very important and require high responsibility from the non-clinical and clinical teams. The available amount of substances could require specific protocols and requirements from the regulatory point of view. For example, the development of vaccines frequently does not require reproductive toxicity, mutagenicity or carcinogenicity tests. On the other hand, each substance has particular characteristics and is developed for the treatment of a specific and complex disease; for this reason, the development program should be analyzed case-by-case. Although the regulatory agencies suggest a basic battery of tests to be performed, it is fundamental that the developmental team anticipates possible additional side effects to elaborate a complete clinic plan with important information and avoiding unnecessary studies. Thus, the previous and direct contact between the pharmaceutical industry and the regulatory agencies is highly recommended, aiming to establish the most appropriate tests for each drug candidate.

### Safety pharmacology

Safety pharmacology is a relatively new area in the process of new drug discovery and development. It started at the end of 1990 with a medical description of severe cardiac side effects with the use of terfenadine (Seldane^®^, Marion Merrell Dow, USA). After thousands of medical prescriptions, it was proven that terfenadine can cause Torsades de Pointes (TdP), which is a lethal cardiac syndrome in healthy subjects caused when terfenadine was used in high doses or in association with other medicines (50). After this incident, the medication was withdrawn from the market. So far, it was believed that only drugs used for cardiac indications could present this severe side effect.

During the development phases of terfenadine, the traditional non-clinical toxicological methods were used, which determined the toxicity of a substance in high doses. However, by using these methods it was not possible to detect the tendency of terfenadine to induce TdP. This problem could have been avoided if, during the routine of the safety tests, a high-throughput screening (HTS) program using biomarkers to TdP had been used in the initial discovery phases of the substance. However, this methodology was not part of the protocols for new drug development. At that time, a specific area of drug development, named safety pharmacology, was created in an attempt to identify the undesirable pharmacodynamic effects of drugs on physiological functions, which are not identified in non-clinical toxicological studies (51).

To determine the risk/benefit rate of a substance in the development phase is particularly difficult when rare, but potentially lethal, side effects are a concern about the new drug (51). In 2001 the ICH approved the guide S7A (52), which requires that the pharmaceutical industries perform battery tests of safety pharmacology to determine potentially undesirable pharmacodynamics effects of a substance, mainly those related to the central nervous system (CNS), cardiovascular and respiratory systems as well as implement supplementary tests to evaluate other systems (53).

Currently, the tendency is that studies of safety pharmacology are not conducted only as a standard battery of tests recommended by the regulatory agencies, but also in an exploratory way in the initial phases of the development process. Thus, with the execution of *in vitro*, *ex vivo*, and *in vivo* preliminary tests of relatively low costs, it is possible to detect early severe side effects allowing a fast remodeling of the data and the reduction of problems related to the safety of a new substance. In addition, such studies help with the decision about continuing the development phase or not. This initial phase is part of the process that supports the selection and optimization of leader candidate substances, in which usually it is not necessary to follow the GLP requirements.

#### Preliminary studies of safety pharmacology

Most of the problems that occur in developmental projects of new drugs or in the withdrawal from the market of an approved new drug are usually associated to cardiovascular safety. Preliminary assays of cardiovascular safety pay special attention to the potential effect of a test article on the cardiac conduction to assess as early as possible whether the drug candidate can induce a delay in the repolarization phase of the ventricular action potential (54). This phenomenon is often associated with the direct block or interruption in the maturation process of the potassium channels hERG (alpha subunit Kv11.1) (55) that are channels of delayed rectification type rapid codified by hERG gene type KCNH2 (56).

The relevance of the hERG channels in the cardiac electric activity became evident after the demonstration that genetic mutations in these channels have been associated with long QT syndrome (LQTS). LQTS is a problem in the electric conduction of the myocardium that alters the ventricular repolarization and, consequently, increases the vulnerability to the development of TdP-type ventricular arrhythmias and the chance of sudden death (57).

Currently, it is well accepted that interference in ventricular repolarization is reflected in the QT interval increase observed in the electrocardiogram, which is the time required for the completion of both ventricular depolarization and repolarization (58). The relationship among the hERG channels inhibition, non-clinical models of the QT interval evaluation, effects on the QT interval in humans, and cardiac arrhythmia is well known and described in the guidelines S7A and S7B (59), which give directions to the evaluation of risk of QT interval prolongation (QT risk). Considering the relevance of these evaluations, it is essential to perform screening for hERG channel inhibition during the process of selection and optimization of the molecule, using the HTS technique. In this context, all of the substances that cause any interference in the hERG channels are considered of potential risk to increase the QT interval. In cases where inhibition of the hERG channels is persistent, the *in silico* modeling is used in association with computational chemistry to help in the medicinal chemistry to redirect the molecule (60).

Besides the hERG assay, at the beginning of each optimization program, the leader substances should be traced in relation to their possible effects on other relevant cardiac ionic channels, such as the L-type calcium channel (Ca_V_1.2), sodium channel (Na_V_1.5) and the channel of delayed rectification type slow (K_v_7.1, I_Ks_) (61–63), since the activation or blocking of such channels can produce pro-arrhythmic events. Other cardiac targets (α and β-adrenergic receptors) should be also evaluated at this stage, as a routine investigation of the off-target effect (60). The cardiovascular safety *in vitro* studies can be supplemented, if necessary, with more sophisticated assays, like the extracellular action potential assay in human embryonic stem cell (ESC)-derived cardiomyocyte (64).

When leader substances are advanced in the optimization phase, cardiac effects could be tested using the heart perfusion test (Langendorff), which provides important information about the electrophysiology, contractile activity and coronary blood flux. A discrete increase in the QT interval could be detected in this model, which is the QT interval prolongation predictive effect in human (60). Thus, the Langendorff test is considered a good method of screening to detect long QT interval in comparison to other methods, such as telemetry in dogs, which fails to detect increases lower than 10% (65). Still in the optimization phase, the test articles could be evaluated on a scale of intravenous doses in anesthetized rats to evaluate effects on the heart frequency and blood pressure. In these evaluations, it is possible to observe dependent dose changes, but further experiments should be performed, including mechanism of action studies or telemetry with awake animals. Also, test articles should be tested in studies with anesthetized dogs that provide additional information about cardiac contractibility, cardiac debit and pulmonary vascular pressure (66). To evaluate possible mechanisms of action, further experiments are often conducted, such as the action potential study in isolated tissues and the study of isolated blood vessels (60). Thus, the global and integrated cardiovascular risk evaluation should consider all of the results obtained *in vitro*, *ex vivo* and *in vivo*.

It is also important to mention that the preliminary safety pharmacology tests for small molecules should not be restricted to the cardiovascular system. Preliminary assays should also evaluate the effect of leader substances on the CNS, respiratory systems, as well as on other systems when necessary. In the exploratory phase, the first *in vivo* test normally executed is the Irwin test (67), which provides rapid detection of the potential toxicity of the test article, the active dose scale and the main actions on the behavior and physiological functions (68). In addition, if the substance is designed to treat CNS diseases or other physiological systems, which have an action on the CNS, it should also be tested in the preliminary studies to evaluate the abuse potential and addiction behavior, in accordance with the “Non-clinical investigation of the dependence potential of medicinal products” (69). The tests to evaluate the susceptibility for abuse are also comprised in the “Abuse Potential of Drugs” (70).

Regarding the respiratory safety determination, *in silico* tests are required to optimize the selected leader substances. These substances are then crossed with a cellular target panel, which has many substances responsible for several respiratory side effects (e.g., contraction/relaxation of the smooth muscle or induction/inhibition of the mucus production). Also, biological assays are performed to identify the activity of the substance on the respiratory system. For more information about relevant cellular targets for respiratory safety tests, please see (51). To confirm further possible actions of the test article in those targets, which could suggest a relevant side effect, it is necessary to determine the action profile, understanding whether it causes inhibition, activation or modulation (54). Indeed, the optimized leader substance can be treated in relation to its pulmonary ventilation and the muscular tone of the respiratory tract using respiratory plethysmography in conscious rats and isolated rat trachea, respectively (54).

#### GLP safety pharmacology studies

The second part of the safety pharmacology program includes a standard test battery defined in guidelines S7A and S7B (52,59). In this phase, the decisions are not based on "excluding substances with potential side effects" but on "presenting a probably safe substance". Thus, there is a change in the development status, in which the regulatory authorities have to decide whether a substance will be evaluated in humans or not.

The guideline S7A (52) describes three types of safety pharmacology studies: a core battery of tests, which includes assessments of vital physiological systems such as CNS, cardiovascular and respiratory systems; supplemental studies, which include more complex physiological systems (gastrointestinal, renal, immune, etc.); and follow-up studies for core battery, which are more detailed and directed to the characterization of specific adverse effects observed in the core battery. On the other hand, the ICH S7B guideline is particularly intended for evaluating the pro-arrhythmic risk of the candidate substance to new medicine.

The essential assays summarized in this set of tests are performed before the phase I clinical trial, using the same route of administration as conventional toxicology studies (usually the same dose that will be used clinically). Assessments are generally conducted for a period of up to 24 h after administration of the test article (51). The battery of recommended tests should be performed in accordance with GLP requirements. Moreover, for the posterior and supplementary tests, there are no specific additional guidelines, although its management should be as close as possible to the GLP.

Importantly, there are conditions where the safety pharmacology studies are not required, such as local agents (dermal and ocular use) and cytotoxic agents for treatment of patients with end-stage cancer (except cytotoxic agents with novel mechanisms of action). For biotechnology-derived products with highly specific binding to the target, it may be sufficient to evaluate the safety pharmacology with toxicology studies and/or pharmacodynamic studies. On the other hand, in biotechnology-derived products that represent a new therapeutic class, or do not have highly specific binding to targets, an extensive safety pharmacology review should be considered.

*Battery tests of the cardiovascular system.* The S7A guideline (52) recommends the monitoring of general cardiovascular parameters. In this context, heart rate, blood pressure (systolic, diastolic and average), ECG parameters and heart morphology are assessed, including tests for the presence of cardiac arrhythmia. For this, the telemetry technique in awake animals is used, which is usually in Latin square design or dose escalation, with complete wash out of the substance considering enough time required between the dosages. These studies often use the same species as toxicology studies (49). Additionally, S7A guideline (52) mentions that assessments of repolarization and conductance abnormalities should be considered. These evaluations are described in more detail in ICH S7B guideline, which is specific to the study of the effect of substances on ventricular cardiac repolarization to determine the pro-arrhythmic risk.

The strategy for the tests described in ICH S7B guideline comprises the *in vitro* evaluation of the substance activity on the hERG channels and *in vivo* on the QT interval. These assays are complementary tools; therefore, both should be conducted. The hERG assay is currently considered a model of choice for evaluation of cardiac pro-arrhythmic risk. Although this assay, performed by binding techniques and automated technology (HTS assay), appears to be appropriate in the early stages (exploratory) of the safety studies, the manual hERG assay is advised for the cardiovascular tests battery. Despite being more laborious, the manual hERG assay is an indicator of function, as opposed to binding technique, which only measures the affinity of the test article to the receptor. Additionally, this method more easily fits the requirements of GLP at this stage of development (71).

The results with the hERG assay cannot be a single standard *in vitro* test conducted for evaluating the pro-arrhythmic risk. Ventricular repolarization is a complex physiological process, which cannot be summarized only in terms of hERG current activity. Agonists of calcium channels, for example, are known agents capable of prolonging the duration of action potential (DPA 90%) and predispose to early and/or late post-depolarization after depolarization, which can lead to TdP (72). Cardiac risk related to this mechanism dependent on calcium cannot be detected by the hERG assay. Furthermore, the use of the hERG assay can lead to incorrect conclusions on cardiac risk, as a partial inhibition of hERG current does not result in prolongation of the DPA-90 because of the compensatory effects of other cardiac ion channels. Thus, to properly evaluate the integrated cardiac risk, further studies should be considered, especially those designed to investigate the electrophysiological properties of the test article, such as evaluation of AP duration using different pro-arrhythmic models (73).

Both the hERG assay and isolated Purkinje nerve fibers are predictive tests, however, there is no *in vitro* technique that can completely reproduce the *in vivo* tests. Thus, as indicated in the regulatory guidelines, the *in vivo* approach in awake animals monitored by telemetry is still an essential component in assessing the pro-arrhythmic risk. Therefore, both *in vivo* and *in vitro* tools should be applied to maximize the chances of an accurate assessment of cardiac risk (74). As described in guideline ICH S7B, the set of results of these studies is part of the integrated risk assessment and support the planning and interpretation of subsequent clinical studies.

*Battery tests of the CNS.* The battery of safety tests on the CNS is composed of simple tests using traditional techniques that can be performed quickly. These tests are often carried out at the beginning of the discovery process of candidate substances to drugs as a form of drug screening to eliminate those with CNS risks. Because of its early application in the safety assessment process, such studies are conducted almost exclusively in rodents (68). Furthermore, neurological assessments can be performed in other species (e.g., in dogs, minipigs or monkeys) (75,76). These studies are generally performed blindly with 10 animals per group (51). The functional observation battery (FOB) (77) and Irwin test (67) can be used to evaluate the effects of a test article on the CNS via motor activity parameters, behavioral changes, coordination, sensory and motor reflex and body temperature. Further studies are related to assessment of the effects on cognitive function (potential for abuse, learning, memory and attention) and brain function (electroencephalogram). Due to the complexity, there are no standard protocols and there is also no requirement that these studies should follow GLP principles (68).

*Battery tests of the respiratory system.* The battery of the safety respiratory system includes simple studies, mostly conducted independently of the toxicological studies, with a single administration or inhalation of the test article, conducted in accordance with GLP requirements (78). They are usually carried out in conscious rodents (in most cases in rats) with eight animals per group given the greater variability of respiratory parameters. Larger animals, such as dogs and monkeys, can also be used when necessary (e.g., if the target is absent in rodents or the pharmacokinetic profile is not appropriate) (51). The S7A guideline (52) suggests performing two series of studies: the test battery and the follow-up studies. The test battery includes quantitative measurements of respiratory rate, tidal volume and hemoglobin oxygen saturation (79). Follow-up studies are needed when there is suspicion of side effects based on the pharmacological properties of the test or when the results of the test battery are indicative of side effects (78). In general, respiratory safety tests include evaluation of the "respiratory pump" efficiency and gas exchange. The ventilatory pattern is evaluated by directly monitoring changes in lung volume and airflow generated by thoracic movements in conscious animals, using plethysmography. Head-out, dual chamber and whole body plethysmography techniques are non-invasive methods that are currently used to evaluate typical parameters of respiration including tidal volume, minute volume and mid-expiratory flow (EF50) (80).

If the core battery indicates, for example, flow limitation by a decrease in EF50 or a rapid shallow breathing pattern, the mechanical properties of the lung can be further evaluated functionally by invasive lung function tests or pulmonary manoeuvres in anesthetized animals using their higher sensitivity and specificity. For the measurement of lung resistance and compliance, a pressure-sensitive catheter is inserted into the pleural cavity or esophagus for the measurement of pleural, airway, or transpulmonary pressure (78).

#### Component interaction between safety pharmacology evaluation and toxicology studies

There is a global tendency to integrate some components of the safety pharmacology studies with toxicology evaluations (81). The development of non-invasive techniques such as ECG monitoring together with respiration, temperature and animal activity, using the external telemetry system, has contributed significantly to this practice (82). This may enhance the overall strategy for risk assessment and has advantages such as increased sensitivity (e.g., increased statistical power) based on the relatively large number of animals used in toxicological studies, reduction of the number of animals needed for safety assessments (in accordance with the guidelines of the NC3Rs), the integration of safety pharmacology data with histopathological and hematological data and cost reduction (52).

As discussed above, currently the safety pharmacology studies are not restricted to running the standard battery of tests designated by S7A and S7B guidelines (52,59) for regulatory submission. The teams involved are committed to developing strategies for early assessment of potential problems related to the safety of the candidate substances using different combinations of tests based on scientific assessments and the particularity of each substance, e.g., in a case-by-case basis. Due to integration into the development process, these strategies not only help in deciding whether to continue the project, but they also guide the discovery teams. These actions lead to the identification of candidate substances with appropriate safety profiles, thereby reducing attrition ratio and enabling a greater chance of success in development.

## Dose transposition from animals to humans

Dose transposing between species involves the use of tools such as allometry, which allows estimation of the safest starting maximum dose for clinical trials usually in healthy volunteers. While the allometric method is of great use in implementing doses, it is not applicable for endogenous hormones and proteins, as well as for the calculation of the maximum dose allowed (83). Furthermore, the physiological and biochemical differences between animal species, such as drug metabolizing, enzymes expression, carriers, among others, should be taken into account; hence, *in silico* methods and PK/PD models are also extensively used as support tools for transposing doses for clinical trials (84,85).

One of the most common mistakes observed in non-clinical studies refers to the dose estimation for human use (Phase I trial) based upon studies carried out in animals (efficacy and safety studies of a new substance). There is a misleading tendency for a linear transposition based on a simple conversion of dose calculation used in small animals (mg/kg), extrapolated to a patient with an average weight of 60 kg. This math causes a huge distortion, however, giving the feeling that the treatment of a large animal would require an exorbitant amount of the test article (86). For proper dose extrapolation from small animals to humans (first dose of the substance in humans) known as human equivalent dose (HED), it is fundamental to consider an important parameter called body surface area (BSA). Table 3 shows the conversion of animal doses to human equivalent doses based on BSA.

**Table:**
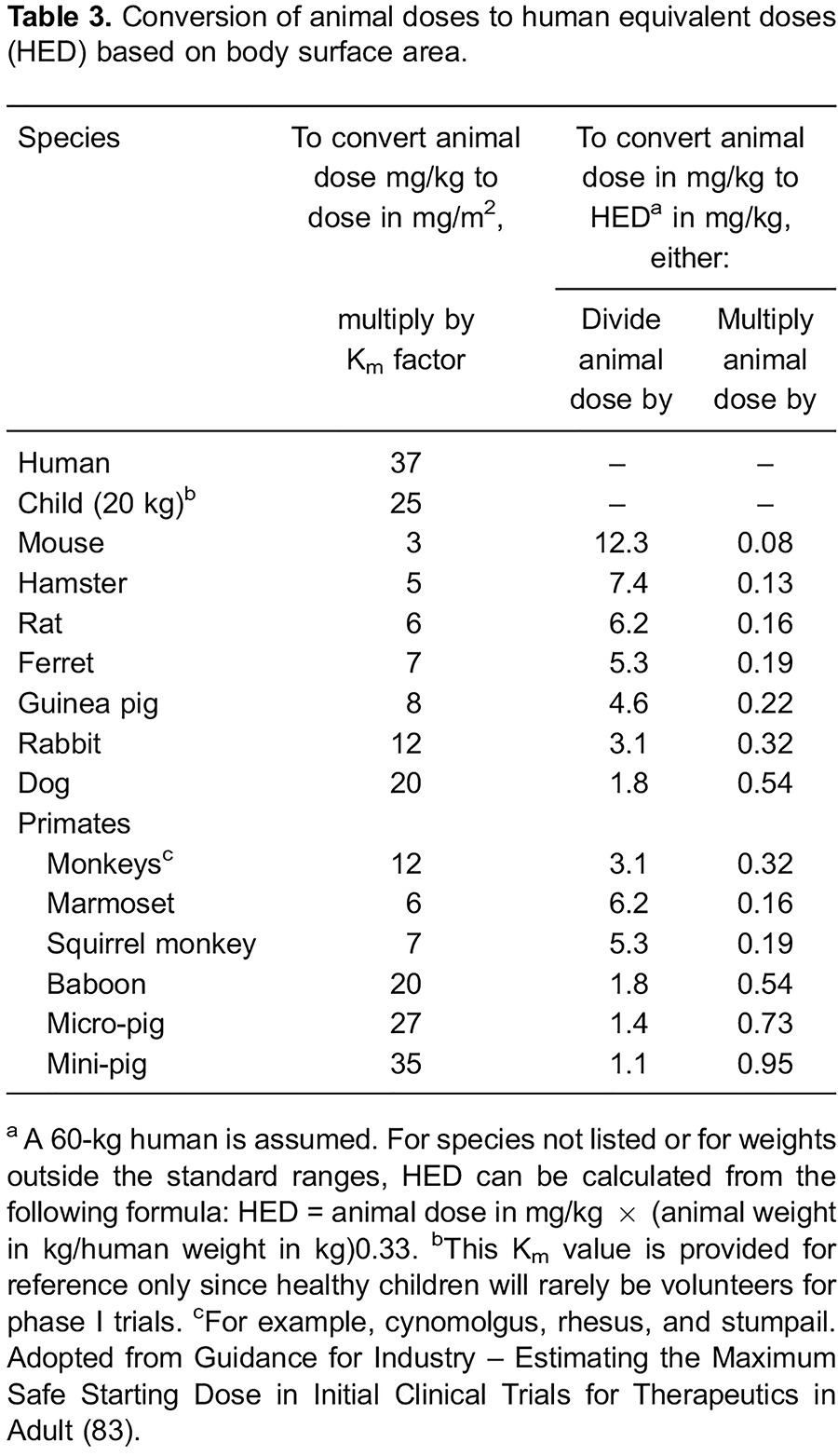

To determine whether HED is mandatory, the previous determination of NOAELs, which can be obtained in the safety (animal toxicology) test, is used. The NOAEL parameter represents the highest dose of tested article in animal specie that does not produce significant adverse effects, as compared to the control group. Thus, to calculate the HED, the dose in animals (mg/kg) should be the NOAEL (87).

## Investigational New Drug Application (IND)

Before starting clinical trials with a new substance, the FDA requires the sponsor/researcher to report on all of the non-clinical studies conducted with the candidate substance for the development of a new medicine as well as the detailed plans for the clinical trials for such product (phases I, II, and III). IND is the mechanism by which the researcher/sponsor informs the FDA about the necessary requirements to receive from the regulatory agency the authorization to initiate the trials in humans (clinical trials). The sponsor/researcher has full responsibility for conducting the clinical studies. The details of the required content and format are described in detail in CFR title 21 part 312 (88) and in the FDA ‘Guidance for Industry: Content and Format of Investigational New Drug Applications (INDs) for Phase 1 Studies of Drugs, Including Well-Characterized, Therapeutic, Biotechnology-derived Products’ (34). Basically, the report for an IND request contains the following information:

• Non-clinical studies results of the test article (*in vitro* and *in vivo*). The non-clinical test checklist may vary according with the product as well as with the clinical trial duration. Furthermore, it must be demonstrated that non-clinical trials were performed in accordance with the GLP requirements;

• Complete chemical information about the new medicine candidate;

• Detailed clinical protocols regarding the Phases I, II, and III experiments, in accordance with the Good Clinical Practices (GCP) rules, as well as other non-clinical studies to be conducted during the research phase in humans (89).

Once the IND application is submitted, the FDA informs the investigator about the reception of documents and it has 30 days to review the data and approve or reject the request. According to the product under development, analysis of the IND request report is performed by the following FDA centers: i) Center for Drug Evaluation and Research (CDER) and ii) Center for Devices and Radiological Health, and Center for Biologics Evaluation and Research (CBER) (90).

## Conclusion and perspectives

In this review, we highlighted the most recent and relevant aspects necessary to conduct non-clinical studies to attend the guidelines to develop new drugs recommended by major regulatory agencies. Although great efforts in recent years have been occurring to reduce, and perhaps in the future, ban the use of animals in the process of new drug development, several alternative methods are being adopted and recommended by the main international regulatory agencies. However, the use of animals in the new drug development process is still required.

Based on this review, it is possible to conclude that there is no single recommended sequence for the achievement of non-clinical studies during the process of new drug development (Figure 1). Many of the studies may be performed in parallel, and the sequence may vary widely depending on the disease. The use of GLP standards is absolutely necessary, especially for the evaluation of safety studies, and is a decisive factor for the acceptance of non-clinical studies in other countries where GLP has been recommended since 1970. Although Brazil adopts practically the same procedures (guidelines) recommended by the FDA and EMA, few laboratories or national institutions can conduct non-clinical studies in accordance with GLP requirements necessary for new drug registration purposes. The lack of reproducibility and reliability of non-clinical studies has been a limiting factor in the process of new drugs development for some national pharmaceutical companies.

Therefore, the need for high quality standard animals, associated with well-designed protocols, qualified human resources, use of positive and negative controls, blind experiment execution, proper use of statistical analyses, among other aspects, are mandatory factors to obtain reliable and reproducible non-clinical results. Non-clinical studies should be strictly performed in accordance with good institutional scientific practices and also employing GLP requirements (indispensable for the request and approval of a IND) in order to ensure the quality, reproducibility and reliability of non-clinical data, which will support the early clinical studies contributing to the successful development of a new drug.

## References

[1] Code of Federal Regulations (CFR) (2015). Title 21. Part 58. Good Laboratory Practice for Nonclinical Laboratory
Studies. Anonymous, Food and Drugs Chapter I.

[2] Code of Federal Regulations (CFR) (1997). Title 40. Part 160. Protection of Environment Chapter I-Environmental
Protection Agency. Anonymous, Good Laboratory Practice Standards.

[3] Code of Federal Regulations (CFR) (2011). Title 40. Part 792. Protection of Environment. Anonymous, Good Laboratory Practice Standards.

[4] Organization for Economic Cooperation and Development (OECD) (1997). Teste Guideline 471: Bacterial Reverse Mutation Test. Anonymous, OECD Guidelines for the Testing of Chemicals.

[5] Handbook Good Laboratory Practice (GLP): quality practices for regulated
non-clinical research and development (2009). World Health Organization (WHO).

[6] Instituto Nacional de Metrologia QeTI (2016). "Cgcre como Autoridade Brasileira de Monitoramento da Conformidade aos
Princípios das Boas Práticas de Laboratório - BPL: Histórico e Adesão è
OCDE".

[7] Instituto Brasileiro do Meio Ambiente e dos Recursos Naturais Renováveis
(IBAMA) (1994). Portaria Normativa No. 139/94.

[8] Instituto Brasileiro do Meio Ambiente e dos Recursos Naturais Renováveis
(IBAMA) (1996). Portaria Normativa No. 84/96.

[9] Instituto Brasileiro do Meio Ambiente e dos Recursos Naturais Renováveis
(IBAMA) (2011). NIT DICLA 035. Princípios das boas práticas de laboratório (BPL).

[10] Seiler JP (2005). Good Laboratory Practice - the Why and the How.

[11] Singh SS (2006). Preclinical pharmacokinetics: an approach towards safer and
efficacious drugs. Curr Drug Metab.

[12] Lee WM (2003). Drug-induced hepatotoxicity. N Engl J Med.

[13] Waring MJ, Arrowsmith J, Leach AR, Leeson PD, Mandrell S, Owen RM (2015). An analysis of the attrition of drug candidates from four major
pharmaceutical companies. Nat Rev Drug Discov.

[14] Roy A (2016). Stifling new cures: the true cost of lengthy clinical drug trials.

[15] Ducharme J, Dudley AJ, Thompson RA, Rang HP (2006). Pharmacokinetic issue in drug discovery. Drug discovery and development.

[16] Meanwell NA (2011). Improving drug candidates by design: a focus on physicochemical
properties as a means of improving compound disposition and safety. Chem Res Toxicol.

[17] Wang J, Urban L (2004). The impact of early ADME profiling on drug discovery and development
strategy. Drug Discov World.

[B18] European Medicines Agency (EMA) (2008). CPMP/EWP/QWP/1401/98: Guideline on the investigation of
bioequivalence.

[B19] Food and Drug Administration (FDA) (2015). Waiver of in vivo bioavailability and bioequivalence studies for
immediate-release solid oral dosage forms based on a Biopharmaceutics
Classification System.

[20] Baranczewki P, Staãczak A, Sundberg K, Svensson R, Wallin A, Jansson J (2006). Introduction to *in vitro* estimation of metabolic s,
IM2tability and drug interactions of new chemical entities in drug discovery and
development. Pharmacol Rep.

[21] Bjornsson TD, Callaghan JT, Einolf HJ, Fischer V, Gan L, Grimm S (2003). The conduct of *in vitro* and *in vivo*
drug-drug interaction studies: a Pharmaceutical Research and Manufacturers of
America (PhRMA) perspective. Drug Metab Dispos.

[B22] Food and Drug Administration (FDA) (2012). Drug interaction studies - study design, data analysis, implications for
dosing, and labeling recommendations.

[B23] European Medicines Agency (EMA) (2012). CPMP/EWP/560/95/Rev. 1: Guideline on the investigation of drug
interactions.

[24] International Conference on Harmonization (ICH) (1994). S3B. Pharmacokinectis: Guidance for repeated dose tissue distribution
studies.

[25] Wienkers LC, Heath TG (2005). Predicting *in vivo* drug interactions from *in
vitro* drug discovery data. Nat Rev Drug Discov.

[26] National Institutes of Health (NIH) (2001). Guidelines for survival bleeding of mice and rats.

[27] Li C, Liu B, Chang J, Groessl T, Zimmerman M, He YQ (2013). A modern *in vivo* pharmacokinetic paradigm: combining
snapshot, rapid and full PK approaches to optimize and expedite early drug
discovery. Drug Discov Today.

[28] International Conference on Harmonization (ICH) (1994). S3A. Note for guidance on toxicokinetics: the assessment of systemic exposure
in toxicity studies.

[29] Buchanan JR, Burka LT, Melnick RL (1997). Purpose and guidelines for toxicokinetic studies within the National
Toxicology Program. Environ Health Perspect.

[30] International Conference on Harmonization (ICH) (2000). Q7: Good manufacturing practiceguide for activeingredient
pharmaceuticals.

[31] International Conference on Harmonization (ICH) (2002). M4Q (R1): The common technical document for the registration of
pharmaceuticals for human use: quality. quality overall summary of module 2 and
module 3.

[32] Food and Drug Administration (FDA) (1995). Guidance for Industry: content and format of investigational new drug
applications (INDs) for phase 1 studies of drugs, including well-characterized,
therapeutic, biotechnology-derived products.

[33] Robinson S, Chapman K, Hudson S, Sparrow S, Spencer-Briggs D, Danks A (2009). Guidance on dose level selection for regulatory general toxicology studies
for pharmaceuticals.

[34] International Conference on Harmonization (ICH) (2009). M3(R2): Guidance on nonclinical safety studies for the conduct of human
clinical trials and marketing authorization for pharmaceuticals.

[35] Organization for Economic Cooperation and Development (OECD) (2008). Teste guideline 407: Repeated dose 28-day oral toxicity study in
rodents. Anonymous, OECD Guidelines for the testing of chemicals.

[36] Organization for Economic Cooperation and Development (OECD) (2014). Test guideline 487: *in vitro* mammalian cell
micronucleus test. Anonymous, OECD Guidelines for the testing of chemicals.

[37] Organization for Economic Cooperation and Development (OECD) (1997). Teste guideline 474: Mammalian erythrocyte micronucleus
test. Anonymous, OECD Guidelines for the testing of chemicals.

[38] Organization for Economic Cooperation and Development (OECD) (1998). Teste guideline 408: Repeated dose 90-day oral toxicity study in
rodents. Anonymous, OECD Guidelines for the testing of chemicals.

[B39] Organization for Economic Cooperation and Development (OECD) (1981). Teste guideline 410: Repeated dose dermal toxicity: 21/28-day
study. Anonymous, OECD Guidelines for the testing of chemicals.

[40] Organization for Economic Cooperation and Development (OECD) (2009). Teste guideline 452: Chronic toxicity studies. Anonymous, OECD Guidelines for the testing of chemicals.

[41] International Conference on Harmonization (ICH) (1998). S4: Duration of chronic toxicity testing in animals (rodent and non-rodent
toxicity testing).

[42] International Conference on Harmonization (ICH) (2005). S5(R2): Detection of toxicity to reproduction for medicinal products and
toxicity to male fertility.

[43] Rang HP, Rang HP (2006). Assessing drug safety. Drug discovery and development.

[44] Agência Nacional de Vigilância Sanitária (ANVISA) (2014). RDC No. 26: Guia de orientação para registro de medicamento fitoterápico e
registro e notificação de produto tradicional fitoterápico.

[45] International Conference on Harmonization (ICH) (1995). S1A: The need for carcinogenicity studies of pharmaceuticals.

[B46] International Conference on Harmonization (ICH) (1997). S1B: Testing for carcinogenicity of pharmaceuticals.

[B47] International Conference on Harmonization (ICH) (2008). S1C(R2): Dose selection for carcinogenicity studies of
pharmaceuticals.

[48] International Conference on Harmonization (ICH) (2005). S8: Immunotoxicity studies for human pharmaceuticals.

[49] European Medicines Agency (EMA) (2000). Guideline on non-clinical local tolerance testing of medicinal products,
draft EMA/CHMP/SWP/2145/2000.

[50] Monahan BP, Ferguson CL, Killeavy ES, Lloyd BK, Troy J, Cantilena LR (1990). Torsades de pointes occuring in association with terfenadine
use. JAMA.

[51] Pugsley MK, Authier S, Curtis MJ (2008). Principles of safety pharmacology. Br J Pharmacol.

[52] International Conference on Harmonization (ICH) (2001). S7A: Guidance for industry: safety pharmacology studies for human
pharmaceuticals.

[53] Pugsley MK (2004). Safety pharmacology matures into a unique pharmacological
discipline. J Pharmacol Toxicol Meth.

[54] Cavero I (2009). Exploratory safety pharmacology: a new safety paradigm to de-risk drug
candidates prior to selection for regulatory science
investigations. Expert Opin Drug Saf.

[55] Dennis A, Wang L, Wan X, Ficker E (2007). hERG channel trafficking: novel targets in drug-induced long QT
syndrome. Biochem Soc Trans.

[56] Warmke JW, Ganetzky B (1994). A family of potassium channel genes related to eag in
*Drosophila and mammals*. Proc Natl Acad Sci USA.

[57] Wisely NA, Shipton EA (2002). Long QT syndrome and anaesthesia. Eur J Anaesthesiol.

[58] QT interval measurements (2002). Card Electrophysiol Rev.

[59] International Conference on Harmonization (ICH) (2005). S7B: The non-clinical evaluation of the potential for delayed ventricular
repolarization (QT interval prolongation) by human pharmaceuticals.

[60] Hornberg JJ, Laursen M, Brenden N, Persson M, Thougaard AV, Toft DB (2014). Exploratory toxicology as an integrated part of drug discovery. Part
II: Screening strategies. Drug Discov Today.

[61] Bodi I, Koch SE, Akhter SA, Schwartz A (2005). The L-type calcium channel in the heart: the beat goes
on. J Clin Invest.

[B62] Harmer AR, Valentin JP, Pollard CE (2011). On the relationship between block of the cardiac Na? channel and
drug-induced prolongation of the QRS complex. Br J Pharmacol.

[63] Towart R, Linders JT, Hermans AN, Rohrbacher J, Van der Linde HJ, Ercken M (2009). Blockade of the I(Ks) potassium channel: an overlooked cardiovascular
liability in drug safety screening?. J Pharmacol Toxicol Methods.

[64] Braam SR, Tertoolen L, van de Stolpe A, Meyer T, Passier R, Mummery CL (2010). Prediction of drug-induced cardiotoxicity using human embryonic stem
cell-derived cardiomyocytes. Stem Cell Res.

[65] Guth BD, Germeyer S, Kolb W, Market M (2004). Developing a strategy for the nonclinical assessment of proarrhythmic
risk of pharmaceuticals due to prolonged ventricular
repolarization. J Pharmacol Toxicol Methods.

[66] Van Deuren B, Van Ammel K, Somers Y, Cools F, Straetemans R, Van der Linde HJ (2009). The fentanyl/etomidate-anaesthetised beagle (FEAB) dog: a versatile
*in vivo* model in cardiovascular safety
research. J Pharmacol Toxicol Methods.

[67] Irwin S (1968). Comprehensive observational assessment: Ia. A systematic, quantitative
procedure for assessing the behavioral and physiologic state of the
mouse. Psychopharmacol.

[68] Castagné V, Froger-Colléaux C, Esneault E, Anne Marie H, Lemaire M, Porsolt RD, Vogel HG, Maas J, Hock FJ, Mayer D (2013). Central nervous system (CNS) safety pharmacology
studies. Drug Discovery and Evaluation: Safety and Pharmacokinetic Assays.

[69] European Medicines Agency (EMA) (2014). EMEA/CHMP/SWP/94227/2014: Guideline on the non-clinical investigation of the
dependence potential of medicinal products.

[70] Food and Drug Administration (FDA) (2010). Guidance for Industry: Assessment of Abuse Potential of Drugs.

[71] Baird TJ, Gaurin DV, Dalton JA, Faqi AS (2013). Contemporary practices in core safety pharmacology
assessments. A comprehensive guide to toxicology in preclinical drug development.

[72] Antzelevitch C, Sicouri S (1994). Clinical relevance of cardiac arrhythmias generated by
afterdepolarizations. Role of M cells in the generation of U waves, triggered
activity and torsade de pointes. J Am Coll Cardiol.

[73] Dumotier BM, Adamantidis MM, Puisieux FL, Bastide MB, Dupuis BA (1999). Repercussions of pharmacologic reduction in ionic currents on action
potential configuration in rabbit Purkinje fibers: are they indicative of
proarrhythmic potential?. Drug Dev Res.

[74] Picard S, Lacroix P (2003). QT interval prolongation and cardiac risk assessment for novel
drugs. Curr Opin Investig Drugs.

[75] Moscardo E, Maurin A, Dorigatti R, Champeroux P, Richard S (2007). An optimised methodology for the neurobehavioural assessment in
rodents. J Pharmacol Toxicol Methods.

[76] Tontodonati M, Fasdelli N, Moscardo E, Giarola A, Dorigatti R (2007). A canine model used to simultaneously assess potential
neurobehavioural and cardiovascular effects of candidate drugs. J Pharmacol Toxicol Methods.

[77] Mattsson JL, Spencer PJ, Albee RR (1996). A performance standard for clinical and functional observational
battery examinations of rats. J Am Coll Toxicol.

[78] Hoymann HG (2012). Lung function measurements in rodents in safety pharmacology
studies. Front Pharmacol.

[79] Hamdam J, Sethu S, Smith T, Alfirevic A, Alhaidari M, Atkinson J (2013). Safety pharmacology - current and emerging concepts. Toxicol Appl Pharmacol.

[80] Gauvin DV, Yoder JD, Dalton JA, Baird TJ (2010). Comparisons of 3 plethysmography techniques for rodent pulmonary
function assessment using ponemah wave-forms analysis. J Pharmacol Toxicol Methods.

[81] Luft J, Bode G (2002). Integration of safety pharmacology endpoints into toxicology
studies. Fundam Clin Pharmacol.

[82] Morton DB, Hawkins P, Bevan R, Heath K, Kirkwood J, Pearce P (2003). Refinements in telemetry procedures Seventh report of the
BVAAWF/FRAME/RSPCA/UFAW Joint Working Group on Refinement, Part A. Lab Animal.

[83] Food and Drug Administration (FDA) (2005). Guidance for Industry - Estimating the Maximum Safe Starting Dose in Initial
Clinical Trials for Therapeutics in Adult Healthy Volunteers.

[84] Shargel L, Wu-Pong S, Yu A (2006). Applied Biopharmaceuticals and Pharmacokinetics.

[85] Leahy DE (2006). Integrating *in vitro* ADMET data through generic
physiologically based pharmacokinetic models. Expert Opin Drug Metab Toxicol.

[86] Boxenbaum H, DiLea C (1995). First-time-in-human dose selection: allometric thoughts and
perspectives. J Clin Pharmacol.

[87] Reigner BG, Blesch KS (2002). Estimating the starting dose for entry into uumans: principles and
practice. Eur J Clin Pharmacol.

[88] Code of Federal Regulations (CFR) (2015). *Title 21. Part 312. Investigational New Drug Application*. Food and Drug Administration Department of Health and Human Services
Subchapter D-Drugs for Human Use.

[88] Food and Drug Administration (FDA) (2015). Investigational New Drug Applications Prepared and Submitted by Sponsor-
investigators - Guidance for Industry - 2015b.

[89] Rolbein ME (2009). Understanding FDA regulatory requirements for investigational new drug
applications for sponsor-investigators. J Investig Med.

